# Lower limb malalignment on whole-leg radiography predicts medial or lateral talar osteochondral lesion location: Implications for osteoarthritis assessment

**DOI:** 10.1016/j.ocarto.2025.100707

**Published:** 2025-11-19

**Authors:** Ralf Henkelmann, Raphael Greifeldt, Pierre Hepp, Robert Hennings, Hans-Jonas Meyer, Timm Denecke, Jeanette Henkelmann

**Affiliations:** a Department for Orthopaedics, Trauma and Plastic Surgery, University Hospital Leipzig, Leipzig, Germany; b Department for Diagnostic and Interventional Radiology, University Hospital Leipzig, Liebigstraße 20, 04103, Leipzig, Germany

**Keywords:** Osteochondritis dissecans, Diagnostic imaging, Talus, Localization, Whole-leg radiographs, Load axis

## Abstract

**Objective:**

To investigate the association between coronal plane malalignment of the lower extremity and talar osteochondritis dissecans (OCD) localization, and assess the influence of mechanical axis deviations and local joint morphology on lesion distribution.

**Design:**

This retrospective monocentric study included 50 patients (mean age 31 ​± ​12 years) with 52 talar OCD lesions who underwent standing whole-leg radiography, which were analyzed digitally. The primary outcome was OCD lesion localization (medial vs. lateral) in relation to coronal plane lower limb alignment (varus, neutral, valgus). Secondary outcomes included angular parameters of the knee and ankle (mLDFA, mMPTA, mLDTA, JLCA, KAJA, talar inclination, talar tilt) and associations lower limb alignment patterns with lesion location. Associations were assessed via univariate testing and binary logistic regression.

**Results:**

Talar OCDs occurred medially in 83 ​% and laterally in 17 ​%. Lower limb malalignment was significantly associated with lesion localization (p ​= ​0.024), with varus correlated with medial, while valgus predicted lateral OCD lesions (OR 2.63; 95 ​% CI 1.1–6.4; p ​= ​0.034). Talar tilt independently correlated with lesion site (p ​< ​0.001); tibiotalar valgus tilt increased odds for lateral lesions (OR 0.084; 95 ​% CI 0.01–0.73; p ​= ​0.025). Combined knee and ankle alignment subtypes also influenced lesion laterality (p ​= ​0.017).

**Conclusions:**

Lower limb malalignment and talar tilt are independent predictors of OCD localization. Coronal plane deviations and joint morphology alter load across the talar dome, contributing to medial or lateral predilection. Whole-leg alignment analysis should be considered in diagnostic and surgical planning, particularly in revision cases.

**Key points**

• Coronal plane malalignment of the lower limb significantly influences the localization of osteochondral lesions in the talus.
• Varus alignment is associated with medial OCD lesions, whereas valgus alignment correlates with lateral lesions.
• Talar tilt, as a surrogate of joint loading asymmetry, independently predicts lesion laterality.
• Whole-leg standing radiographs provide essential diagnostic information beyond local ankle imaging.
• Preoperative assessment of limb alignment should be considered in all patients with talar OCD to optimize joint-preserving strategies and reduce the risk of cartilage degeneration.

## 1 Introduction

Osteochondritis dissecans (OCD) of the talus is a rare but clinically relevant condition, predominantly affecting adolescents and young adults, with an estimated incidence of 4.6 per 100,000 [[1], [2], [3]]. In the ankle joint, OCD lesions most commonly affect the medial and central aspects of the talar dome, followed by lateral compartments [4,5]. Despite extensive investigation, the etiology remains multifactorial and incompletely understood, with vascular insufficiency, repetitive microtrauma, and altered mechanical loading considered key contributing factors [[6], [7], [8]].

While biomechanical alignment is a well-established determinant of OCD localization in the knee—where varus and valgus deformities correlate with medial and lateral lesion distribution, respectively - a comparable relationship in the ankle has not been clearly defined [9,10]. Previous studies have suggested that talar morphology and joint alignment are associated with load distribution within the ankle joint [11,12]. However, the role of whole-leg mechanical axis deviation (MAD) in determining the localization of talar OCD lesions remains largely unexplored.

Given the growing understanding of osteoarthritis as a disease in association with mechanical environment and joint congruity, clarifying these relationships is crucial—not only for pathophysiological insight but also for improving diagnostics, risk stratification, and surgical planning. We hypothesize that leg malalignment—specifically varus or valgus deviation—alters local joint loading patterns and contributes to the preferential occurrence of OCD lesions on the medial or lateral talar dome, respectively.

Thus, the primary research question of this study is whether coronal plane alignment deviations of the lower extremity, as assessed on standing whole-leg radiography, are associated with the localization of talar OCD lesions, and how this information can inform clinical decision-making and treatment strategies.

## 2 Method

This retrospective monocentric study includes all patients diagnosed with talar OCD between 2013 and 2024 ​at Leipzig University Hospital who underwent standing whole-leg radiography. Inclusion criteria were [1] confirmed image-based diagnosis of OCD of the talar dome, and [2] availability of standing whole-leg radiography meeting predefined quality criteria. The study was approved by the local ethics committee (185/25-ek) and performed in accordance with the Declaration of Helsinki. The requirement for informed consent was waived in accordance with §34 of the Saxon Hospital Law.

In addition to epidemiological data, the defect localization on the talus was determined from the radiographs and classified according to the International Cartilage Repair Society (ICRS) guidelines [13].

Conventional standing whole-leg anteroposterior radiography of both lower extremities were obtained in a standardized position with patients standing upright and facing forward. To ensure reproducibility of the mechanical axis assessment, image acquisition adhered to predefined quality criteria: centrally positioned patella, symmetrical projection of the femoral condyles, tibiofibular overlap of approximately one-third, and inclusion of the entire limb from femoral head to ankle joint. Radiographs not meeting these criteria were excluded. A radiographic marker was used for calibration.

The primary outcome was the localization of the talar osteochondral lesion (medial vs. lateral dome) in relation to coronal lower limb alignment (varus, neutral, valgus). Secondary outcomes included angular parameters of the knee and ankle (mLDFA, mMPTA, mLDTA, JLCA, tibial curvature, KAJA, talar inclination, talar tilt), associations between combined knee and ankle alignment patterns and lesion location, and exploratory correlations between radiological alignment parameters and available clinical data.

Radiographs were analyzed using validated software (mediCAD 2D, version 7.0.0.3, Hectec GmbH, Germany, and IDS 7, version 25.1, Sectra Workstation, Sweden) by a single board-certified radiologist specialized in musculoskeletal imaging, following standardized protocols for image analysis. Measurement of mechanical and anatomical parameters was then independently reviewed by an experienced orthopedic and trauma surgeon with subspecialization in joint surgery to ensure clinical plausibility and anatomical accuracy.

The mechanical axis of the lower extremity was assessed via established angular parameters, including: mechanical lateral proximal femoral angle (mLPFA), mechanical lateral distal femoral angle, mechanical medial proximal tibial angle, mechanical lateral distal tibial angle, joint line convergence angle, MAD.

Neutral alignment was defined according to Paley (mLDFA and mechanical medial proximal tibial angle between 85° and 90°; mechanical lateral distal tibial angle 86°–92°) [14]. Deviations above or below these thresholds indicated varus or valgus alignment, respectively. In cases of compensatory deformity, opposing angulations at the knee and ankle could result in an overall neutral alignment.

To further differentiate alignment contributions, the knee and ankle joints were classified independently as varus, neutral, or valgus, resulting in nine possible combinations. These alignment subgroups were used for categorical analysis.

**Fig. 1:**
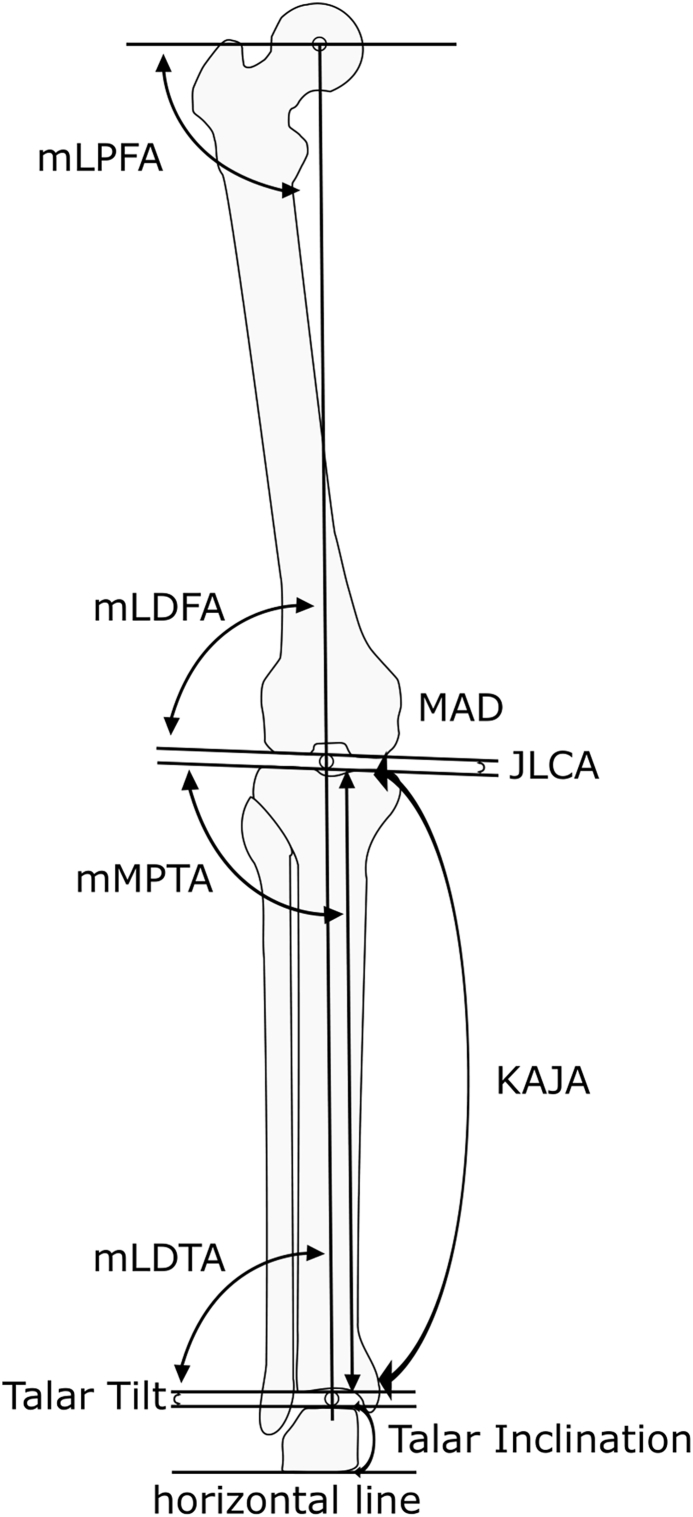
Schematic standing whole-leg radiograph illustrating the measured radiological parameters. Mechanical lateral proximal femoral angle (mLPFA), mechanical lateral distal femoral angle, mechanical medial proximal tibial angle, mLDTA, joint line convergence angle, MAD, knee-ankle joint line angle (KAJA).

In addition to mechanical axis analysis, anatomical parameters were assessed, including tibial curvature in the frontal plane, the knee–ankle joint line angle (KAJA), and talar inclination (TI) [15,16]. Talar tilt (TT), defined as the angle between the tibial plafond and the talar dome. Negative talar tilt angles were defined as laterally opening, indicating medial joint space narrowing, whereas positive angles corresponded to tibiotalar valgus tilt, reflecting lateral joint space narrowing. Fig. 1 illustrates the measured parameters.

Descriptive statistics were calculated for all variables. Normality was assessed using the Shapiro-Wilk test. Group comparisons were performed using Student's t-test or Fisher's exact test, as appropriate. Binary logistic regression was applied to determine associations between variables and binary outcomes, reported as odds ratios with 95 ​% confidence intervals (CI). A p-value <0.05 was considered statistically significant. Analyses were conducted using IBM SPSS Statistics, version 29 (IBM Corp., Armonk, NY, USA). Given the retrospective design, a post-hoc power analysis was performed for the association between lower limb alignment and OCD lesion localization. Based on the observed effect size (Cramer's V ​= ​0.36, n ​= ​52), the achieved power was 0.82, indicating that the sample size was sufficient to detect a significant association at α ​= ​0.05.

## 3 Results

A total of 1372 patients with the following ICD10 codes were screened: M19.17, M19.27, M19.97, M24.07, M24.17, M93.27, M93.87. After confirmation of OCD at the head of the talus, 426 patients remained in the collective. These were further examined for doublets and the presence of a standing whole-leg radiography with corresponding quality criteria.

A total of 50 patients (46 ​% male) with 52 OCD of the talus were included, with a mean age of 31 ​± ​12 years (range, 10–55). Based on ICRS classification, 13.5 ​% of lesions were grade 2, 63.5 ​% grade 3, and 23.1 ​% grade 4. The mean duration of symptoms prior to diagnosis was 26 ​± ​34 months (range, 0–180). Surgical intervention was performed in 81 ​% of cases, and 33 ​% of patients underwent revision surgery.

**Fig. 2:**
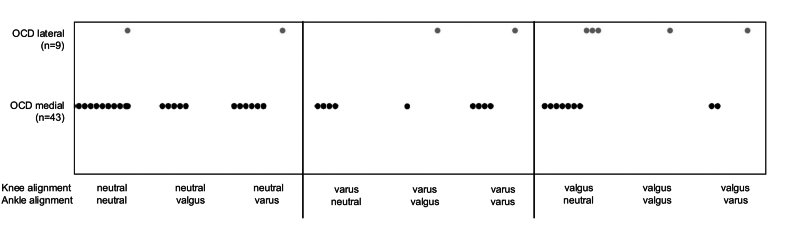
Distribution of medial and lateral talar osteochondritis dissecans (OCD) across combined knee and ankle alignment patterns, defined by combinations of mLDFA, mMPTA, and mLDTA. Each point represents one OCD lesion classified by alignment profile and localization.

Regarding the primary outcome, OCD lesions were localized medially in 83 ​% with right-sided occurrence in 52 ​%. Mechanical limb alignment was significantly associated with lesion localization (medial vs. lateral) (p ​= ​0.024), indicating that overall coronal plane alignment is associated to the distribution of lesions. Patients with valgus alignment exhibited significantly higher odds of having lateral OCD lesions compared to medial ones (OR 2.63; 95 ​% CI 1.1–6.4; p ​= ​0.034), confirming lower limb malalignment as an independent predictor of lesion laterality. For secondary outcomes, combined knee and ankle alignment patterns, defined by mLDFA, mMPTA, and mLDTA, were significantly related to lesion laterality (p ​= ​0.017), and the distribution of the nine combined alignment subgroups is illustrated in Fig. 2.

**Fig. 3:**
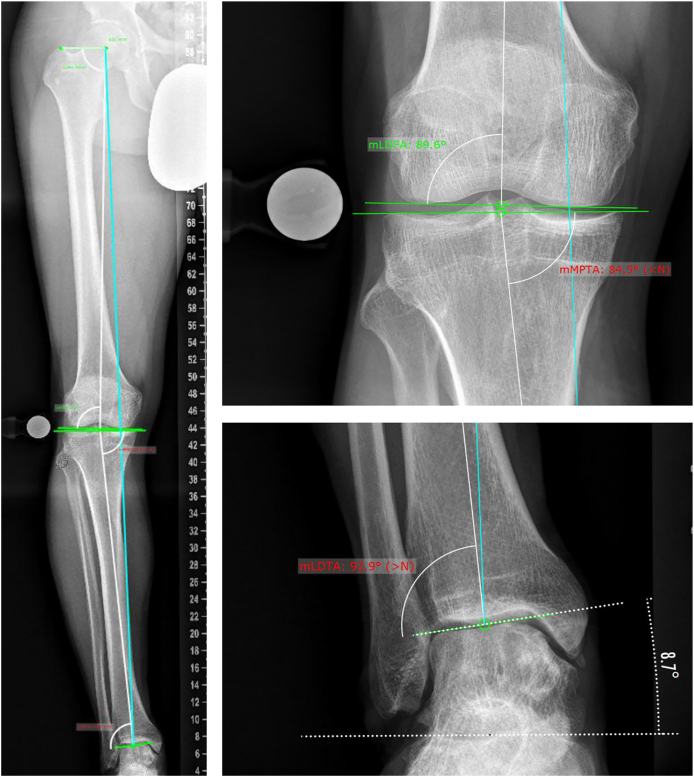
Standing whole-leg radiography demonstrating axial malalignment of the lower limb with varus deformity at the knee and upper ankle joint, accompanied by pathological talar inclination.

Fig. 3 shows a representative case presenting with a medial osteochondral lesion of the talus accompanied by knee varus, ankle varus, and pathological TI. Talar tilt was independently correlated with lesion location (p ​< ​0.001); specifically, a tibiotalar valgus tilt increased the odds for lateral OCD lesions (OR 0.084; 95 ​% CI 0.01–0.73; p ​= ​0.025). Binary logistic regression further confirmed these findings, demonstrating a significant overall effect of multi-joint alignment configurations on OCD distribution (OR 1.4; 95 ​% CI 1.1–1.9; p ​= ​0.027), suggesting that specific knee and ankle alignment patterns are associated with the localization of talar OCD.

Table 1, Table 2 summarize the associations between mechanical alignment parameters, talar tilt, and lesion localization, supporting the primary and secondary outcomes as described. Specific multi-joint alignment configurations influence the distribution of OCD lesions.

**Table 1:** Association between lower limb alignment and OCD lesion location (medial vs. lateral talar dome).

Alignment category	OCD medial (%)	OCD lateral (%)	*p*^1^	OR (95 ​% CI)	*p*^2^
**Mechanical limb alignment**					
Neutral	58.2	22.2	0.024∗	2.6 (1.1–6.4)	0.034∗
Varus	20.9	22.2			
Valgus	20.9	55.6			
**Combined knee and ankle alignment**					
Neutral/Neutral	32.6	11.1	0.017∗	1.4 (1.04–1.9)	0.027∗
Neutral/Varus	11.6	0			
Neutral/Valgus	14.0	11.1			
Varus/Neutral	9.3	0			
Varus/Varus	2.3	11.1			
Varus/Valgus	9.3	11.1			
Valgus/Neutral	16.3	33.4			
Valgus/Varus	0	11.1			
Valgus/Valgus	4.6	11.1			

Mechanical limb alignment refers to the mechanical axis of the lower extremity (hip–knee–ankle). Knee and ankle alignments were assessed separately and analyzed in combination to form subgroups. p^1^: univariate comparison; ​p^2^: binary logistic regression. OR ​= ​odds ratio; CI ​= ​confidence interval. Significant results marked with ​p ​< ​0.05.∗.

**Table 2:** Radiographic ankle parameters in relation to OCD lesion location (medial vs. lateral talar dome).

Ankle	OCD medial mean ​± ​SD	OCD lateral mean ​± ​SD	p^1^	OR (95 ​% CI)	p^2^
KAJA (°)	1.3 ​± ​4.7	−0.1 ​± ​5.9	0.7	1.04 (0.83–1.31)	0.71
Talar tilt (°)	−1.3 ​± ​1.5	1.1 ​± ​1.1	<0.001∗	0.08 (0.01–0.7)	0.02∗
Talar Inklination (°)	−1.7 ​± ​5.6	−3.4 ​± ​5.2	0.4	1.02 (0.84–1.24)	0.85

Values are presented as mean ​± ​standard deviation. p^1^: univariate comparison; p^2^: binary logistic regression. OR ​= ​odds ratio; CI ​= ​confidence interval. Significant results marked with p ​< ​0.05.∗.

## 4 Discussion

The aim of this study was to investigate whether a correlation exists between the localization OCD of the talus and mechanical alignment deviations of the lower limb based on standing whole-leg radiography. Our findings reveal a significant association between medially located talar OCD and varus alignment, and between laterally located OCD and valgus alignment. These results support the hypothesis that MAD influences the pressure distribution within the ankle joint and may contribute to the development or localization of OCD.

A detailed analysis of coronal alignment demonstrated that a varus leg axis correlates with medial talar OCD, while valgus alignment is linked to lateral OCD. This corresponds with biomechanical studies showing that the medial aspect of the talus typically bears higher pressure loads [10,11]. Bruns et al. described a medial predilection for osteochondral lesions due to consistently elevated pressure in that region [17].

The direction of mechanical limb alignment (varus, neutral, valgus) appears to influence the vector of joint loading across the talar dome, with varus alignment consistently associated with medial pressure concentration and valgus alignment with lateral. In neutral alignment, OCD localization was variable. Notably, lateral OCDs coincided more frequently with a laterally directed presumed pressure focus than medial OCDs did with a medial one.

The impact of mechanical realignment on the ankle joint has been addressed in several previous studies. Kim et al. reported increased ankle pain in patients with knee osteoarthritis undergoing medial open-wedge high tibial osteotomy, noting compensatory hindfoot changes [18]. Similarly, Oh et al. demonstrated a shift in the load axis after HTO, supporting our observation that varus alignment leads to increased medial pressure, which can be laterally redirected postoperatively [19]. Krause et al. identified changes in ankle joint pressure following knee-proximal osteotomy in cadaveric models, while Suero et al. showed that progressive valgisation of the lower limb alters ankle joint pressure distribution by decreasing contact area and increasing focal stress [20,21].

Our stepwise biometric analysis revealed that talar tilt, reflecting localized pressure alterations, was significantly associated with the development and localization of talar OCD. Specifically, a tibiotalar valgus tilt was present in all patients with lateral OCD lesions, independent of overall varus or valgus limb alignment. These findings, consistent with previous studies demonstrating that coronal plane deviations of the tibia–talus complex affect ankle loading and OCD distribution, highlight that both global limb alignment and local joint morphology are associated with lesion localization and may inform individualized surgical planning [22].

In our cohort, the direction of talar tilt corresponded with OCD lesion location, suggesting a compensatory adaptation of TI to altered joint loading. Prior studies have associated increased talar tilt with more severe chondral damage and larger OCD lesions in the context of chronic lateral ankle instability [[23], [24], [25]]. Additionally, varus tibial alignment has been linked to the progression of ankle osteoarthritis, highlighting the association to alignment-related stress on osteochondral pathology [26].

This study highlights the critical role of assessing lower limb alignment in patients with talar OCD, particularly prior to surgical intervention. Our findings reveal a clinically significant correlation between lower limb malalignments and the localization of talar OCD lesions, emphasizing the need for precise evaluation of both mechanical and anatomical alignment to optimize treatment outcomes. In our cohort, a revision surgery rate of 35 ​% was observed, underscoring the complexity of managing OCD in the presence of limb malalignment. While current literature reports revision rates averaging around 15 ​%, with some series describing rates as high as 50 ​%, our data suggest that unrecognized biomechanical factors—such as limb alignment—may contribute to suboptimal outcomes in select patients [[27], [28], [29]]. This high revision rate indicates that restoring optimal biomechanics extends beyond mere axis correction and requires careful consideration of joint loading adaptations and potential secondary degenerative changes in the ankle joint. Consequently, standing whole-leg radiography should be obtained—at minimum during the planning of revision surgery—in patients with osteochondral lesions of the ankle to allow for a comprehensive assessment of lower limb alignment. In select cases, corrective osteotomy may serve as a biomechanically sound adjunct to joint-preserving strategies, potentially mitigating cartilage degeneration and improving long-term joint function in the context of osteoarthritis and OCD.

Clinically, these results emphasize the importance of evaluating whole-leg alignment in patients with talar OCD, as this assessment can clarify lesion localization and explain persistent or recurrent symptoms. In select cases, mechanical axis correction — such as high tibial or distal femoral osteotomy — may be integrated into an individualized treatment plan, taking into account patient-specific factors and surgeon judgment, as practice patterns may vary. Importantly, in revision cases of talar OCD, a comprehensive whole-leg analysis should be performed to identify underlying malalignment that may contribute to recurrent or therapy-resistant lesions. Alignment assessment during preoperative planning may further optimize joint-preserving procedures and reduce the risk of secondary ankle degeneration.

This study has several limitations, including the rarity of talar OCD, which restricted cohort size, the retrospective design with potential selection bias, and underrepresentation of ICRS grade II lesions, as imaging is typically performed after symptom onset. Inter- and intra-observer reliability was not assessed; however, all measurements were performed by an experienced observer using validated software and standardized protocols. Despite these limitations, the study's strengths include standardized whole-leg radiography for comprehensive coronal alignment assessment and detailed correlation with talar OCD localization. The incorporation of alignment evaluation into diagnostic and preoperative planning may facilitate individualized therapeutic strategies and optimize biomechanical load distribution. Moreover, given the rarity of talar OCD, the cohort is comparatively robust and demonstrates a clear trend in alignment-associated lesion localization, highlighting the need for larger, multicenter studies with extended follow-up to validate and expand these findings.

In conclusion, lower limb malalignment and talar tilt are independent determinants of medial versus lateral talar osteochondral lesion localization. Incorporating comprehensive whole-leg alignment assessment into diagnostic evaluation and preoperative planning may facilitate individualized therapeutic strategies and thereby optimize biomechanical load distribution.

## Author contributions

R.H. and J.H. conceived and designed the study, collected and analyzed data, and drafted the manuscript. R.G. contributed to data collection and analysis. R.G. and H.-J.M. provided writing support and contributed to critical revision of the manuscript. R.H., P.H. and T.D. critically revised the manuscript for important intellectual content.

## Declaration of Generative AI in scientific writing

Generative AI tools were used solely for language editing, including grammar, sentence structure, and readability. All scientific content, data analysis, interpretation, and conclusions presented in this manuscript are the original work of the authors.

## Role of the funding source

Supported by the Open Access Publishing Fund of 10.13039/501100008678Leipzig University.

## Conflict of interest

The authors declare no competing interests.
